# Public Awareness on Cord Blood Banking in Saudi Arabia

**DOI:** 10.1155/2018/8037965

**Published:** 2018-04-03

**Authors:** Dunia Jawdat, Sulaiman AlTwijri, Hadeel AlSemari, Mayssa Saade, Ahmed Alaskar

**Affiliations:** ^1^Cord Blood Bank, King Abdullah International Medical Research Center, Ministry of National Guard Health Affairs, Riyadh, Saudi Arabia; ^2^College of Medicine, King Saud bin Abdulaziz University for Health Sciences, Ministry of National Guard Health Affairs, Riyadh, Saudi Arabia; ^3^Dental Colleges, King Saud University, Riyadh, Saudi Arabia; ^4^Biostatistics and Modeling Section, King Abdullah International Medical Research Center, Ministry of National Guard Health Affairs, Riyadh, Saudi Arabia; ^5^Department of Oncology, King Abdulaziz Medical City, Ministry of National Guard Health Affairs, Riyadh, Saudi Arabia

## Abstract

**Background:**

In the last decade, cord blood (CB) has proven to be a valuable source of hematopoietic stem cells for transplantation to treat many hematological disorders. Since then, many CB banks have been established worldwide. Our aim was to estimate the level of public awareness of CB banking in Saudi Arabia.

**Study Design and Methods:**

A self-administered questionnaire of 22 multiple choices was conveniently distributed, consisting of demographics, awareness measure, attitude toward banking preference, and donation for research data.

**Results:**

A total of 1146 participants have completed the questionnaire. The majority were young female 19–25 years old (26%), who are college graduates (57%) with middle class socioeconomic status (82%). The subjective assessment of the overall knowledge was inadequate (66%). For the objective assessment, 12 questions were asked about CB source, collection, storage, and usage. Only half of the subjects (52%) knew that CB is a source of stem cells. More than half did not know the main use of CB. About half did not know about the method of collection nor the condition of storing.

**Conclusion:**

This study shows a high lack of knowledge about CB banking. More than half of the subjects were unaware of CB banking and its uses. However, most subjects are accepting CB storage, which anticipates great impact and efficacy on educational programs. Moreover, the data demonstrated that health professionals were not the source of knowledge. We recommend having comprehensive educational campaigns with clear information about CB banking to facilitate positive perspectives towards donation and scientific research.

## 1. Introduction

While the umbilical cord blood (UCB) was considered medical waste, this is no longer the case. Umbilical cord blood is now widely accepted as a valuable source of hematopoietic stem cells (HSCs), serving as an alternative source to bone marrow and peripheral blood HSCs [1]. Since the first cord blood transplantation, the usage of UCB has been growing as a viable treatment option in hematologic and oncologic disorders with comparable results to bone marrow and peripheral blood transplantations [2].

Today, hematopoietic stem cell transplantation (HSCT) is used to treat many disorders including acute [3] and chronic leukemia [4], myelodysplastic syndromes [5], *β* thalassemia [6], aplastic anemia, Fanconi anemia [7], bone marrow failures, immune deficiencies [8], and metabolic diseases [7]. UCB also contains mesenchymal stem cells (MSCs), endothelial progenitor cells, and immune cells—all of which can unveil possible novel treatment modalities for many untreated diseases.

UCB is not routinely preserved; it is done upon request. Hence, individuals should be informed of the once in a lifetime opportunity to collect and preserve UBC for the variety of treatment modalities it can offer; be it for the individual, their families, or the general public. It is of high importance to assess the level of knowledge about CB banking in our community, as well as the accuracy of the information people receive, as the quality of information will highly impact the decision of parents, on whether to incinerate their child's UCB or store it in a bank. Misleading information with unrealistic hope about the future usage of CB will also influence the type of bank, whether public or private, that parents would choose.

Today, more than 700,000 UCB units are stored in public cord blood banks (CBBs) worldwide for any patient in need for stem cell transplantation. Saudi Arabia has currently 2 public CBBs and no private banks, as they are prohibited by law. However, both public banks offer related banking for families with history of hematological disorder. Also, several private companies offer their collection services to families, which prefer to store their UCB in private banks and ship the UCB to international storage facilities.

A number of studies have been conducted in several countries to learn about peoples' knowledge, understanding, preferences, and attitude toward CB banking. Unfortunately, most results have shown inadequate knowledge in the public, expecting mothers and even medical staff or health care providers to know about it [9–15]. Herein, we aimed to estimate the level of awareness of CB banking in the public of Saudi Arabia.

## 2. Materials and Methods

The study was approved by the local research ethics committee. The participation was voluntary; the only exclusion was people unwilling to answer the questionnaire. Data was collected by using a self-administered questionnaire. The questionnaire consisted of three major sections: (1) demographics, (2) awareness measure, and (3) attitude determinant. The first section included information related to the sample demographics, such as age, education level, and socioeconomic level. The second section included questions about CB banking and its uses. The third determined the attitude toward CB banks, from willingness to store UCB to bank model preference.

Raw data was processed in accordance with best practices for raw data management to identify any inaccuracies or incompleteness in advance to the statistical analysis. In order to accomplish this task, all interval variables were checked and summarized in terms of maximum and minimum values. Minimum and maximum values were checked and compared against the nominal maximum and minimum values of each variable, and variables with implausible values were flagged. A similar process will be applied to categorical variables to identify any potential anomalies (e.g., miscodes) by conducting frequency analysis. All identified were addressed accordingly.

All study variables were summarized and reported across the study subjects using descriptive statistics. Categorical variables (age, educational level, socioeconomic status, and attended clinic) were summarized and reported in terms of frequency distribution. Univariate descriptive analysis was used to summarize the overall knowledge of CB banking among participants. Results were reported in terms of frequency, percent, and 95% confidence interval. The same analysis was also applied to summarize public knowledge and attitude towards CB banking. Results were reported in terms of count and percent. All analyses were conducted using SAS version 9.4 (SAS Institute Inc., Cary, NC).

## 3. Results

A total of 1146 participants have completed the questionnaire. The majority of the participants were female (88%), 19–25 years old (26%), who are college graduates (57%) with middle class socioeconomic status (82%) as presented in Table 1. The subjective assessment of the overall knowledge about CB banking was poor, as the majority (66%) assessed their level of knowledge to be inadequate, while 29% judged to be satisfactory and 5% superior as shown in Table 2. For the objective assessment, 12 questions were asked about CB, in terms of source, collection, storage, and usage. Only half of the subjects (52%) knew that CB is a source of stem cells. More than half of the population surveyed did not know the main use of CB. Also, about half of the participants did not know about the method of collection nor the condition of storing as shown in Table 3. When we compared the answers of males to females, we found that in the subjective assessment, 65.7% of the females versus 69.5% of the males assessed their overall knowledge about cord blood banking as inadequate. Also, in the objective assessment, more females answered the questionnaire correctly compared to males, in all areas including cord blood source, collection, storage, and usage (data not shown).

When subjects were asked about the source of information, the response was social media for most participants (51%), followed by traditional media (25%), hospital educational materials (14%), and medical personnel (10%) as presented in Table 4. When subjects were asked about their choice between public and private banks, more than three quarters (80%) preferred public donation to private storage of cord blood. Finally, when subjects were asked to bank cord blood for research purposes, if their donated CB does not meet clinical criteria, 88% supported its use in research. As regards the decision of cord blood donation, 87% believed that it should be shared between future parents as shown in Table 4.

## 4. Discussion

The purpose of this study was to establish the overall awareness and attitude of CB banking within the Saudi Arabian population. A total of 1146 individuals were surveyed to determine the knowledge and attitude towards CB banking. The majority (66%) of the participants assessed their knowledge as inadequate. For the objective assessment, 12 questions were asked about CB source, collection, storage, and usage. The results showed poor overall knowledge, as more than 40% had 0–4 correct responses, while only 23% had 9–12 correct responses.

Unfortunately, poor knowledge about CB banking has been seen in many countries [9, 14, 15]. A previous study conducted in 5 European countries showed that 79% of the surveyed subjects had poor knowledge about CB banking [10]. Similar results were seen in a more recent study conducted in India where only 26% of the participants knew what CB banking meant [11]. A survey in Greece showed that 48% of the participants had full knowledge about CB donation and storage [12]. Better awareness of CB banking was seen in Australia where 70% indicated that they were aware of CB banking [13].

The majority of the participants (51%) who had heard about cord blood banking were informed mainly through social media, and only 10% were informed by medical staff—this was also seen in other countries [10]. This could be due to insufficient knowledge of the medical staff themselves not allowing them to lead confident discussion about CB banking as seen in some studies [16]. It is very important to educate our medical staff or health care providers with accurate and detailed information about CB banking and the current therapeutic use, as they are on the front line and most people prefer getting such information from their health care givers. Getting information from social media alone may give misleading information that may negatively affect people's decision on whether to donate their cord blood. Providing educational programs to medical staff will improve the awareness of patients and their families from a trusted source with no financial interest.

Interestingly, the attitude of the participants was positive towards CB donation and most of them (80%) preferred public donation to private storage, if given the option. This attitude will help in expanding our pool of CB donors for allogeneic stem cell transplantation. Surprisingly, 88% of the participants, if given the option, are willing to donate their CB for research if not suitable for clinical use. This will aid in the development of new treatments and improvements in the field of stem cell transplantation. Regarding the decision of CB donation, 87% believed that both parents should be included in the decision whether to donate and store their child CB. This was also seen in other countries [10]. We highly recommend having comprehensive educational campaigns with clear and accurate information about CB banking to the public in Saudi Arabia, to facilitate positive perspectives towards CB donation and scientific research.

## Figures and Tables

**Table 1:** Demographic characteristics of the population studied.

Demographic characteristic	Category	Total = 1146, number (%)
Gender	Male	(131) 11.4%
	Female	(1015) 88.6%
Age	19–25	(301) 26.3%
	26–30	(121) 10.6%
	31–35	(160) 14%
	36–40	(161) 14%
	41–45	(188) 16.4%
	Over 45	(215) 18.8%
Education	Elementary, middle, or high school graduate	(377) 32.9%
	College graduate	(656) 57.2%
	Postgraduate degree	(113) 9.9%
Socioeconomic status	Low	(22) 1.9%
	Middle	(934) 81.5%
	High	(190) 16.6%

**Table 2:** Overall subjective knowledge analysis.

Demographic characteristic	Category of assessment	Total = 1146, number (%)	Lower confidence limit	Upper confidence limit
Knowledge	Superior	(60) 5.2%	0.039	0.065
	Satisfactory	(328) 28.6%	0.259	0.313
	Inadequate	(758) 66.1%	0.633	0.689

**Table 3:** Objective knowledge about cord blood banking.

Information and knowledge questions	Answers	Total = 1146, number (%)
Cord blood is	Blood in cord blood after birth	(220) 19.2%
	Blood in placenta after birth	(173) 15.1%
	Both	(221) 19.3%
	I do not know	(532) 46.4%
Umbilical cord blood can provide a rich source of	Proteins	(46) 4%
	Vitamins	(31) 2.7%
	Stem cells	(601) 52.4%
	I do not know	(468) 40.8%
Cord blood collection is done	Before delivery	(59) 5.1%
	After delivery	(710) 62%
	I do not know	(377) 32.9%
In case of no donation, cord blood is always	Given to parents	(11) 1%
	Medical waste	(880) 76.8%
	I do not know	(255) 22.3%
Cord blood can be collected from	Natural births	(96) 8.4%
	Cesarean sections	(34) 3%
	Both	(600) 52.4%
	I do not know	(416) 36.3%
Cord blood collection is painless for the mother and the baby	True	(629) 54.9%
	False	(34) 3%
	I do not know	(483) 42.1%
Are there any health risks associated with cord blood collection?	Yes	(50) 4.4%
	No	(552) 48.2%
	I do not know	(544) 47.5%
Cord blood can treat diseases such as	Bone fractures	(20) 1.7%
	Blood cancer	(494) 43.1%
	Epilepsy	(9) 0.8%
	I do not know	(623) 54.4%
Cord blood infusion can treat the same diseases as a bone marrow transplant	True	(373) 32.5%
	False	(35) 3.1%
	I do not know	(738) 64.4%
Cord blood is stored for many years at	Room temperature	(82) 7.2%
	Extremely low temperature	(394) 34.4%
	I do not know	(670) 58.5%
Who is the beneficiary of the stored cord blood?	Cord blood-donated child	(145) 12.7%
	Any match	(396) 34.6%
	Research	(194) 16.9%
	I do not know	(411) 35.9%
Cord blood can be stored for	1 year	(60) 5.2%
	5 years	(48) 4.2%
	20 years	(146) 12.7%
	I do not know	(892) 77.8%

**Table 4:** Knowledge and attitude analysis towards cord blood banking.

Questions	Answers	Total = 1146, number (%)
What has been your major source of information on the subject?	Hospital educational material	(165) 14.4%
	Medical personnel	(110) 9.6%
	Traditional media	(288) 25.1%
	Social media	(583) 50.9%
Where would you store your cord blood?	Public cord blood bank	(912) 79.6%
	Private cord blood bank	(234) 20.4%
Would you donate for research?	Yes	(1002) 87.4%
	No	(144) 12.6%
Should the decision about donation be shared between parents?	Yes	(998) 87.1%
	No	(148) 12.9%

## Supplementary Materials

Supplementary Materials Table 1: subjective knowledge analysis by gender. Table 2: objective knowledge about cord blood banking by gender. Table 3: objective knowledge about cord blood banking by gender.
