# Supplementary material for: Public Awareness on Cord Blood Banking in Saudi Arabia
Source: Stem Cells Int. 2018 Apr 3;2018:8037965. doi: 10.1155/2018/8037965 (PMC5903187; doi:10.1155/2018/8037965)
Supplement: Supplementary Materials — Table 1: subjective knowledge analysis by gender. Table 2: objective knowledge about cord blood banking by gender. Table 3: objective knowledge about cord blood banking by gender. [file 8037965.f1.docx]

**Table 1. Subjective Knowledge Analysis by Gender**

| Demographic Characteristic | Category of assessment | Male  (n=131) | Female  (n=1015) |  |
| --- | --- | --- | --- | --- |
| Knowledge | Superior | (11) 8.40 % | (49) 4.83 % |  |
|  | Satisfactory | (29) 22.14 % | (299) 29.46 % |  |
|  | Inadequate | (91) 69.47 % | (667) 65.71 % |  |

**Table 2. Objective Knowledge about cord blood banking by gender**

| Information and knowledge Questions | Answers | Male  (n=131) | Female  (n=1015) |  |
| --- | --- | --- | --- | --- |
| Cord blood is | Blood in cord blood after birth | (19)14.50% | (201)19.80% |  |
|  | Blood in placenta after birth | (13)9.92% | (160)15.76% |  |
|  | Both | (23)17.56% | (198)19.51% |  |
|  | I don’t know | (76)58.02% | (456)44.93% |  |
| Umbilical cord blood can provide a rich source of | Proteins | (2)1.53% | (44)4.33% |  |
|  | Vitamins | (3)2.29% | (28)2.76% |  |
|  | Stem cells | (66)50.38% | (535)52.71% |  |
|  | I don’t know | (60)45.80% | (408)40.20% |  |
| Cord blood collection is done | Before delivery | (7)5.34% | (52)5.12% |  |
|  | After delivery | (67)51.15% | (643)63.35% |  |
|  | I don’t know | (57)43.51% | (320)31.53% |  |
| In case of no donation cord blood is always | Given to parents | (1)0.76% | (10)0.99% |  |
|  | Medical waste | (81)61.83% | (799)78.72% |  |
|  | I don’t know | (49)37.40% | (206)20.30% |  |
| Cord blood can be collected from | Natural births | (18)13.74% | (78)7.68% |  |
|  | Cesarean sections | (5)3.82% | (29)2.86% |  |
|  | Both | (42)32.06% | (558)54.98% |  |
|  | I don’t know | (66)50.38% | (350)34.48% |  |
| Cord blood collection is painless for mother and baby | True | (64)48.85% | (565)55.67% |  |
|  | False | (1)0.76% | (33)3.25% |  |
|  | I don’t know | (66)50.38% | (417)41.08% |  |
| Are there any health risks associated with cord blood  collection? | Yes  No | (9)6.87%  (50)38.17% | (41)4.04%  (502)49.46% |  |
|  | I don’t know | (72)54.96% | (472)46.50% |  |
| Cord blood can treat diseases such as | Bone fractures | (2)1.53% | (18)1.77% |  |
|  | Blood cancer | (49)37.40% | (445)43.84% |  |
|  | Epilepsy | (5)3.82% | (4)0.39% |  |
|  | I don’t know | (75)57.25% | (548)53.99% |  |
| Cord blood infusion can treat the same diseases as a  bone marrow transplant | True  False | (41)31.30%  (12)9.16% | (332)32.71%  (23)2.27% |  |
|  | I don’t know | (78)59.54% | (660)65.02% |  |
|  |  |  |  |  |
| Cord blood is stored for many years at | Room temperature | (9)6.87% | (73)7.19% |  |
|  | Extremely low temperature | (45)34.35% | (349)34.38% |  |
|  | I don’t know | (77)58.78% | (593)58.42% |  |
| Who is the beneficiary of the stored cord blood? | Cord blood donated child | (14)10.69% | (131)12.91% |  |
|  | Any match | (35)26.72% | (361)35.57% |  |
|  | Research | (27)20.61% | (167)16.45% |  |
|  | I don’t know | (55)41.98% | (356)35.07% |  |
| Cord blood can be stored for | 1 year | (6)4.58% | (54)5.32% |  |
|  | 5 years | (11)8.40% | (37)3.65% |  |
|  | 20 years | (9)6.87% | (137)13.50% |  |
|  | I don’t know | (105)80.15% | (787)77.54% |  |

**Table 3. Objective Knowledge about cord blood banking by gender**

| Information and knowledge Questions | Male  (n=131) | Female  (n=1015) | p-value |
| --- | --- | --- | --- |
| Cord blood is |  |  |  |
| *Incorrect Other* | *108(82.44%)* | *817(80.49%)* | *0.6397* |
| *Correct Both* | *23(17.56%)* | *198(19.51%)* |  |
| Umbilical cord blood can provide a rich source of |  |  |  |
| *Incorrect Other* | *65(49.62%)* | *480(47.29%)* | *0.6427* |
| *Correct Stem cells* | *66(50.38%)* | *535(52.71%)* |  |
| Cord blood collection is done |  |  |  |
| *Incorrect Other* | *64(48.85%)* | *372(36.65%)* | *0.0075** |
| *Correct After delivery* | *67(51.15%)* | *643(63.35%)* |  |
| In case of no donation cord blood is always |  |  |  |
| *Incorrect Other* | *50(38.17%)* | *216(21.28%)* | *<0.0001** |
| *Correct Medical waste* | *81(61.83%)* | *799(78.72%)* |  |
| Cord blood can be collected from |  |  |  |
| *Incorrect Other* | *89(67.94%)* | *457(45.02%)* | *<0.0001** |
| *Correct Both* | *42(32.06%)* | *558(54.98%)* |  |
| Cord blood collection is painless for mother and baby |  |  |  |
| *Correct TRUE* | *64(48.85%)* | *565(55.67%)* | *0.1616* |
| *Incorrect Other* | *67(51.15%)* | *450(44.33%)* |  |
| Is there any health risks associated with cord blood collection? |  |  |  |
| *Incorrect Other* | *81(61.83%)* | *513(50.54%)* | *0.0157** |
| *Correct* | *50(38.17%)* | *502(49.46%)* |  |
| Cord blood can treat diseases such as |  |  |  |
| *Incorrect Other* | *82(62.60%)* | *570(56.16%)* | *0.1893* |
| *Correct Blood cancer* | *49(37.40%)* | *445(43.84%)* |  |
| Cord blood infusion can treat the same diseases as a  bone marrow transplant |  |  |  |
| *Correct* | *41(31.30%)* | *332(32.71%)* | *0.7674* |
| *Incorrect Other* | *90(68.70%)* | *683(67.29%)* |  |
| Cord blood is stored for many years at |  |  |  |
| *Incorrect Other* | *86(65.65%)* | *666(65.62%)* | *1* |
| *Correct Extremely low temperature* | *45(34.35%)* | *349(34.38%)* |  |
| Who is the beneficiary of the stored cord blood? |  |  |  |
| *Incorrect Other* | *96(73.28%)* | *654(64.43%)* | *0.0507* |
| *Correct Any match* | *35(26.72%)* | *361(35.57%)* |  |
| Cord blood can be stored for |  |  |  |
| *Incorrect Other* | *122(93.13%)* | *878(86.50%)* | *0.0357** |
| *Correct 20 years* | *9(6.87%)* | *137(13.50%)* |  |

**Fisher’s exact test*
